# Combining 16S rRNA Sequencing and Metabolomics Data to Decipher the Interactions between Gut Microbiota, Host Immunity, and Metabolites in Diarrheic Young Small Ruminants

**DOI:** 10.3390/ijms241411423

**Published:** 2023-07-13

**Authors:** Xinlu Wang, Lili Niu, Yaxuan Wang, Siyuan Zhan, Linjie Wang, Dinghui Dai, Jiaxue Cao, Jiazhong Guo, Li Li, Hongping Zhang, Tao Zhong

**Affiliations:** 1 Farm Animal Genetic Resources Exploration and Innovation Key Laboratory of Sichuan Province, Sichuan Agricultural University, Chengdu 611130, China; wangxinlu1996@163.com (X.W.); niulili@sicau.edu.cn (L.N.); siyuanzhan@sicau.edu.cn (S.Z.); wanglinjie@sicau.edu.cn (L.W.); 71317@sicau.edu.cn (D.D.); jiaxuecao@sicau.edu.cn (J.C.); jiazhong.guo@sicau.edu.cn (J.G.); zhp@sicau.edu.cn (H.Z.); 2 Key Laboratory of Livestock and Poultry Multi-Omics, Ministry of Agriculture and Rural Affairs, College of Animal and Technology, Sichuan Agricultural University, Chengdu 611130, China

**Keywords:** ruminants, immune, metabolome, serum biochemistry, 16S rRNA

## Abstract

Diarrhea is associated with gut microbiota, immunity, and metabolic alterations in goat kids and lambs. This study used 28 lambs (11 healthy and 17 diarrheic) and 20 goat kids (10 healthy and 10 diarrheic) to investigate the association between diarrhea occurrence and changes in gut microbiota, metabolism, and immunity in goat kids and lambs. The results revealed that Firmicutes, Proteobacteria, and Bacteroidetes were the dominant phyla in goat kids and lambs. In addition, *Enterobacteriaceae* and *Lachnospiraceae* families were identified in both diarrheic goat kids and lambs. Furthermore, functional prediction of microbiota showed that it was involved in cell motility and cancer pathways. The identified differential metabolites were implicated in the bile secretion pathway. Lambs had significant differences in immunoglobulin G (IgG), immunoglobulin M (IgM), interleukin-1β (IL-1β), and tumor necrosis factor-alpha (TNF-α) compared to goat kids. IgG and IL-1β were positively correlated to Patescibacteria, *Clostridiaceae*, and unclassified_Muribaculaceae in both diarrheic goat kids and lambs. In addition, weighted gene co-expression network analysis (WGCNA) revealed that the MEgreen module was positively associated with IgG, IgM, IL-1β, TNF-α, and triglyceride (TG). In conclusion, our results characterized the gut microbiota, metabolism, and immune status of lambs and goat kids suffering from diarrhea.

## 1. Introduction

The occurrence of diarrhea is common in young small animals, which negatively affects their growth and development and even leads to death [1,2]. Following intensive and large-scale husbandry development, sheep farms with tens or hundreds of thousands are springing up. Refined feeding management and scheduled vaccination could reduce the incidence of bacterial and viral diarrhea. However, changes in diet transition, artificial weaning, and metabolic disorders are the leading causes of diarrhea in young lambs and goats [2,3]. Accumulating evidence indicates that diarrhea has multiple effects on the host, such as altering the steady state of gut microbiota, intestinal mucosa pathological changes, and alterations in several enzyme activities [4]. In turn, gut microbial dysbiosis may affect animal health, increasing susceptibility to many diseases and the occurrence of diarrhea [5,6]. Therefore, it is necessary to investigate the interaction between the occurrence of diarrhea and changes in gut microbiota, immunity, and metabolic status.

Previous studies found that the gut microbiota is a complex ecosystem that regulates immunity, maintains the intestinal mucosal barrier, and contributes to digesting host food, especially absorbing free fatty acids [7,8]. Several studies have shown that *E. coli*, rotavirus, and Cryptosporidium parvum are commonly the causative agents of diarrhea in young animals, including calves [9], lambs [10], and pigs [11]. Studies in lambs and goat kids reported that diarrhea is associated with gut microbiota alterations [12,13,14]. For instance, the proportion of dominant bacteria such as Firmicutes, Proteobacteria, and Bacteroidetes varied to some extent [12,14]. The relative abundance of *Fusobacteria*, *Actinobacteria*, *Verrucomicrobia*, and Proteobacteria also changes in diarrheic piglets [15]. *Commensal Butyricicoccus*, *Faecalibacterium*, *Ruminococcus*, *Collinsella*, and *Coriobacterium* were indexes of the health status of calves [16].

Most studies proposed that diarrhea and gut microbiota imbalance are mutually causal [17], and adjusting the balance of gut microbial may play a positive role in treating diarrhea. In recent years, some progress has been triggered in acknowledging the regulation of gut flora health and improving the immunity of young animals. For instance, dietary *Bacillus amyloliquefaciens-9* and quercetin supplementation enhance the immunity status of young animals [18,19]. Furthermore, fecal microbiota transplantation (FMT) regulates gut microbiota and reduces the incidence of diarrhea to a certain extent [20,21,22]. FMT could increase *Porphyromonadaceae* in the intestine, decrease fecal amino acid concentration, and relieve diarrhea symptoms in calves [23]. Moreover, feeding probiotics, such as GBacillus-9 and *Bacillus amyloliquefaciens-9*, is also an effective way to improve animal immunity and the microbial community structure of the gastrointestinal tract [24,25].

The occurrence of diarrhea also modifies blood biomarkers in young ruminants. The contents of immunoglobulin G (IgG) and immunoglobulin M (IgM) are reduced in lambs suffering from diarrhea [3]. Additionally, the albumin concentration decreased in calves suffering from diarrhea, and the globulin and haptoglobin contents differed from healthy ones [26]. In fact, immunoglobulin Y (IgY) antibody treatment can effectively prevent the occurrence of diarrhea [27]. The changes in serum protein concentration, pro-inflammatory cytokines, and other substances with health status also reflect the microbiota function [26,28]. The gut microbiota plays a vital role in the nutrient metabolism of animals [29,30], producing metabolites, such as short-chain fatty acids associated with the synthesis of immune proteins [31]. *Ruminococcaceae*, *Caldicoprobacteraceae*, *Coriobacteriaceae*, and *Eggthellaceae* are involved in the degradation of cellulose polysaccharides and the metabolism of flavonoids [32]. Although the potential influence of diarrhea on growth, immunity, and metabolism has been confirmed in livestock animals, there is a lack of studies comparing the impact of the incidence of diarrhea in the said aspects between different species of small ruminants. The aim of this study was to compare the composition, function, and metabolism of the gut microbiota of lambs and goat kids suffering from diarrhea.

## 2. Results

### 2.1. Diarrhea Alters the Diversity and Composition of the Fecal Microbiota of Lambs and Goat Kids

**Figure 1:**
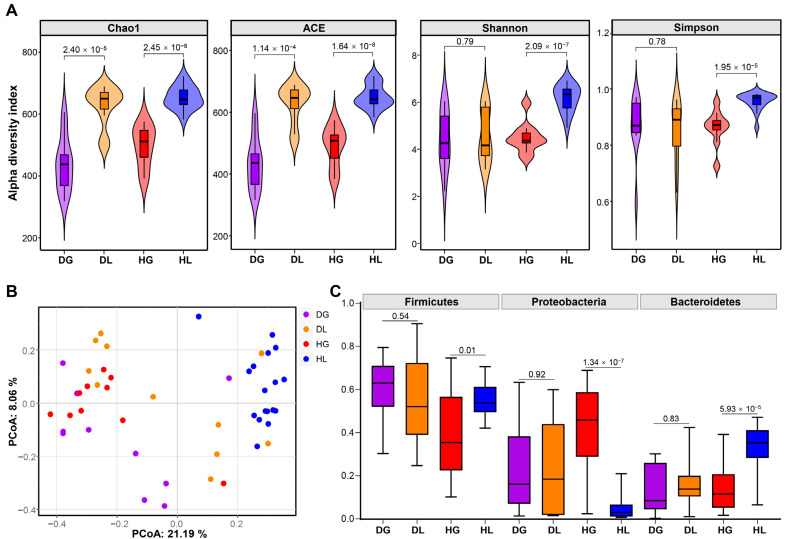
Alpha diversity indices and beta diversity analysis in goat kids and lambs. (**A**) Alpha diversity indices, including ACE, Chao1, Shannon, and Simpson. (**B**) Weighted UniFrac distance-based beta diversity analysis. (**C**) Dominant phylum on healthy and diarrheic goat kids and lambs. DG: diarrheic goat kids, DL: diarrheic lambs, HG: healthy goat kids, HL: healthy lambs.

A total of 866 OTUs were identified, including 15 phyla, 21 classes, 59 orders, 108 families, and 242 genera (Supplementary Materials Table S1). The fecal bacterial community diversity in diarrheic and healthy lambs and goat kids was determined using 16S rRNA sequencing. The alpha and beta diversity of the microbiota among DG, HG, DL, and HL groups were compared using the observed species richness index. Chao1 and ACE significantly differed between goat kids and lambs regardless of health status (Figure 1A). However, in the Shannon and Simpson index, there was no difference between goat kids and lambs (Figure 1A). Regarding the beta diversity, the results showed differences in the fecal microbiota composition among groups (Figure 1B). Firmicutes, Proteobacteria, and Bacteroides were significantly different between HG and HL groups. However, there were no significant differences between DG and DL groups in the abundance of Firmicutes, Proteobacteria, and Bacteroidetes. In addition, regardless of health status, Firmicutes, Proteobacteria, and Bacteroidetes were dominant in goat kids and lambs (Figure 1C).

**Figure 2:**
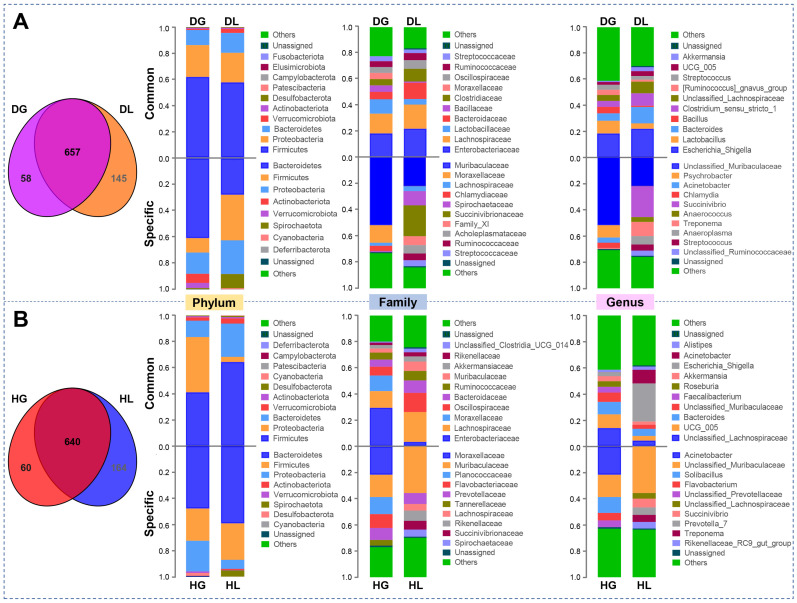
The Venn diagram shows the number of bacterial OTUs (at 97% identity) of the top 10 fecal microbiota shared and unique by different groups of goat kids and lambs. (**A**) Diarrheic goat kids and lambs. (**B**) Healthy goat kids and lambs. For abbreviations, see Figure 1.

The Venn plot indicated that 657 shared operational taxonomic units (OTUs) between DL and DG groups (Figure 2A). In parallel, DL had 145 unique OTUs, while DG had a fewer number of OTUs (58). At phylum levels, Firmicutes, Proteobacteria, Bacteroidetes, Actinobacteriota, and Verrucomicrobiota emerged as shared and unique bacteria. At the family level, *Streptococcaceae*, *Ruminococcaceae*, *Lachnospiraceae*, and *Moraxellacea* were both shown as shared and unique. However, the shared and unique OTUs did not have any same bacteria at the genus level (Figure 2A). The Venn diagram displayed that 640 OTUs were shared between HG and HL groups. Simultaneously, HG goat kids had 60 unique OTUs, while HL lambs had more OTUs (164, Figure 2B). At the phylum level, more phylum was annotated by the common OTUs. *Rikenellaceae*, *Muribaculaceae*, *Moraxellaceae*, and *Lachnospiraceae* were presented as the shared and unique bacteria at the family level. In addition, only *Acinetobacter* was detected as the shared and unique bacteria at the genus level (Figure 2B). We further analyzed the bacteria shared by goat kids and lambs regardless of health status. In diarrheic states, only Patescibacteria was significantly different at the phylum level. However, many bacteria differed in HG and HL groups, including Firmicutes, Proteobacteria, Bacteroidetes, *Enterobacteriaceae*, *Oscillospiraceae*, *Muribaculaceae*, *Escherichia_Shigella*, unclassified_Lachnospiraceae, and *UCG_005* (Supplementary Materials Figure S1).

### 2.2. Diarrhea Shifts of Enriched Genera and Their Potential Functions

**Figure 3:**
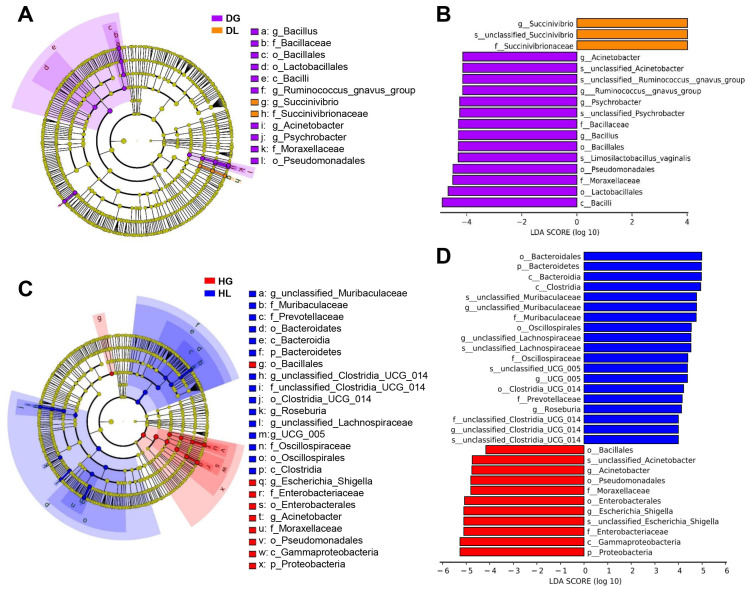
Differences in gut microbiota in different health states of goat kids and lambs. Taxonomic cladogram derived from LEfSe analysis (**A**) and histogram of Linear discriminant analysis (LDA) > 4 for differentially abundant genera (**B**) between DG (purple) and DL (orange). Taxonomic cladogram (**C**) and histogram (**D**) between HG (red) and HL (blue). For abbreviations, see Figure 1.

Linear discriminant analysis effect size (LEFSe) was used to determine the differences in bacterial composition between lambs and goat kids under different health states. *Bacillus*, *Moraxellaceae*, Bacillaceae, *Psychrobacter*, *Acinetobacter, Ruminococcus_gnavus*, and Lactobacillales were enriched in DG goat kids, whereas the abundance of *Succinivibrio* and *Succinivibrionaceae* were enriched in DL lambs (Figure 3A,B). Moreover, Bacteroidetes, *Muribaculaceae*, *Oscillospiraceae*, *Prevotellaceae*, *UCG_005*, and *Roseburia* were enriched in HL lambs. However, Proteobacteria, *Moraxellaceae*, *Enterobacteriaceae*, *Acinetobacter*, and *Escherichia_Shigella* were enriched in HG goat kids (Figure 3C,D).

**Figure 4:**
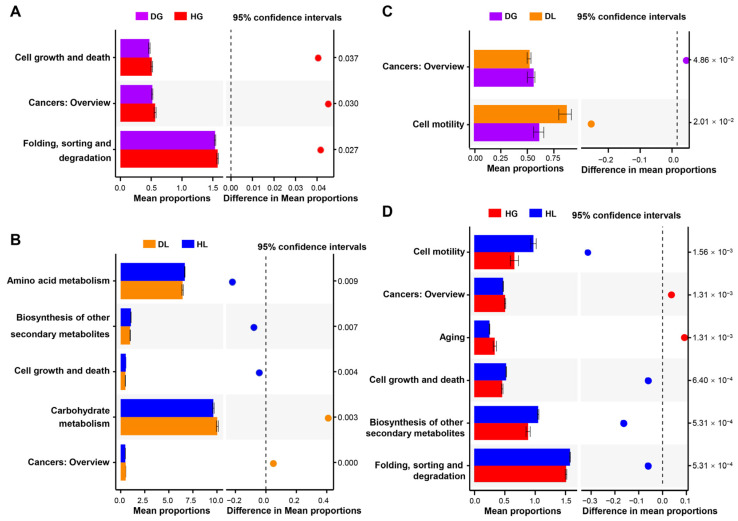
Prediction of the differential functions of fecal microbiota based on PICRUSt 2 in goat kids and lambs. (**A**) Diarrheic versus healthy goat kids. (**B**) Diarrheic versus healthy lambs. (**C**) Diarrheic goat kids versus diarrheic lambs. (**D**) Healthy goat kids versus healthy lambs. *p* was calculated based on Fisher’s exact test.

We studied the association between gut microbiome function and lamb health status by sequencing data from 16S rRNA genes. Kyoto Encyclopedia of Genes and Genomes (KEGG) functional predictions indicated that their annotations significantly differed between DG and HG groups, including cell growth and death, cancers, folding, sorting, and degradation (Figure 4A). Additionally, five annotations had significant differences between DL and HL groups, including amino acid metabolism, biosynthesis of other secondary metabolites, cell growth and death, carbohydrate metabolism, and cancers (Figure 4B). Only cell motility and cancers significantly differed between DG and DL groups (Figure 4C). However, there were more annotations with significant differences between HG and HL groups: cell motility, cancers, aging, cell growth and death, biosynthesis of other secondary metabolites, folding, sorting, and degradation (Figure 4D).

### 2.3. Diarrhea Changes Biochemical Blood Indexes in Lambs and Goat Kids

Regardless of health status, the immune indexes interleukin-1β (IL-1β), tumor necrosis factor-alpha (TNF-α), IgG, IgM, albumin (Alb), and total cholesterol (TC) were significantly different between lambs and goat kids. Total protein (TP), triglyceride (TG), and blood urea nitrogen (BUN) were significantly different between DL and DG, while glucose (Glu) blood concentration did not differ. The TP and Glu blood concentrations were significantly different between HL and HG groups, while BUN did not differ (Table 1).

### 2.4. Diarrhea Alters Serum Metabolism in Both Lambs and Goat Kids

**Figure 5:**
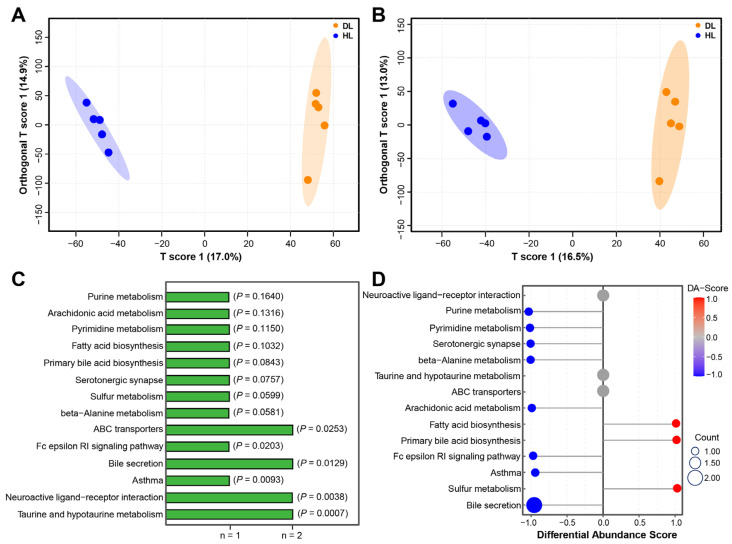
Serum metabolome changes between DL and HL. PLS-DA of metabolite composition in positive (**A**) and negative ion modes (**B**), respectively. (**C**) Pathway enrichment analysis was performed using significantly different metabolites. (**D**) Differential abundance scores of differential metabolic pathways using significantly different metabolites.

We performed the non-targeted metabolomic analysis of lambs with different health states to determine their serum metabolic profiles. Using partial least squares discriminant analysis (PLS-DA), we observed that QC samples were tightly clustered together in positive and negative ion modes, indicating the reliability of the experiment (Figure 5A,B). We identified 276 metabolites, including 171 in positive mode and 105 in negative mode (Supplementary Materials Table S2). VIP > 1 and *p* < 0.05 were set as the standard for screening differential metabolites with a significant difference. Nineteen metabolites were found in positive and negative ion modes. Furthermore, KEGG analysis of all differential metabolites showed that they enriched in 14 metabolic pathways (Figure 5C). Subsequently, a differential abundance score for each pathway was calculated to explore the overall metabolite changes in these pathways. Most metabolic pathways were reduced. Nevertheless, three pathways were increased in DL lambs (Figure 5D). As for goat kids, our previous studies have shown that more metabolites and differential metabolites were identified. In addition, KEGG analysis showed that the pathways were less in goat kids than in lambs. Moreover, these pathways were mainly reduced, with only a few increases in goat kids suffering from diarrhea [12]. Significant metabolites in both lambs and goat kids were enriched in the bile secretion pathway.

### 2.5. Associations of Serum Biochemical Indexes with Microbiota and Differential Metabolites

**Figure 6:**
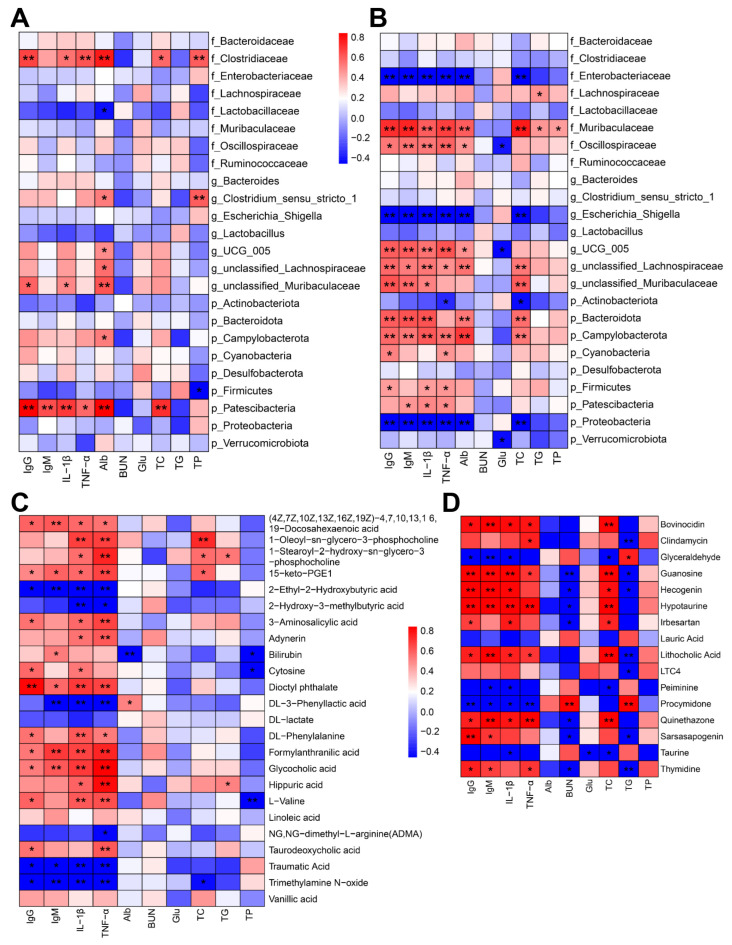
Correlation heatmap of serum biochemical indexes with metabolism and microorganisms. Correlation analyses between serum biochemical indexes and microbiota on goat kids and lambs suffering from diarrhea (**A**) and healthy goat kids and lambs (**B**). Correlation analyses between serum biochemical indexes and differential metabolites on goat kids (**C**) and lambs (**D**). * *p* < 0.05; ** *p* < 0.01.

IgG and IL-1β concentration were positively correlated to Patescibacteria, *Clostridiaceae*, and unclassified_Muribaculaceae content in diarrheic animals. Furthermore, Alb concentration was positively related to more bacteria, including Patescibacteria, Campylobacterota, unclassified_Lachnospiraceae, unclassified_ Muribaculaceae, *UCG_005*, *Clostridium_sensu_stricto_1*, and *Clostridiaceae*. However, Firmicutes were negatively correlated with TP concentration (Figure 6A). Interestingly, more bacteria were significantly associated with IgG, IgM, IL-1β, and TNF-α in healthy individuals (Figure 6B). IgG, IgM, IL-1β, and TNF-α were positively correlated with many bacteria, including Campylobacterota, *Muribaculaceae*, *Oscillospiraceae*, *UCG_005*, and unclassified_Lachnospiraceae. Conversely, all four immune indexes were negatively related to *Escherichia_Shigella* (Figure 6B). Furthermore, all the immune indexes were positively correlated with (4Z,7Z,10Z,13Z,16Z,19Z)-4, 7, 10, 13, 16, 19-Docosahexaenoic acid, 15-keto-PGE1, Dioctyl phthalate, Formylanthranilic acid, and Glycocholic acid in goat kids. However, 2-Ethyl-2-Hydroxybutyric acid, Taurodeoxycholic acid, and Trimethylamine N-oxide were negatively correlated with IgG, IgM, IL-1β, and TNF-α (Figure 6C). As for lambs, all four immune indexes were positively correlated with five metabolites (Bovinocidin, Guanosine, Hypotaurine, Lithocholic acid, and Quinethazone). Conversely, only Peiminine was negatively correlated with IgG, IgM, IL-1β, and TNF-α (Figure 6D).

### 2.6. Hub Microbiota Associated with Serum Factors on Diarrheic Goat Kids and Lambs

**Figure 7:**
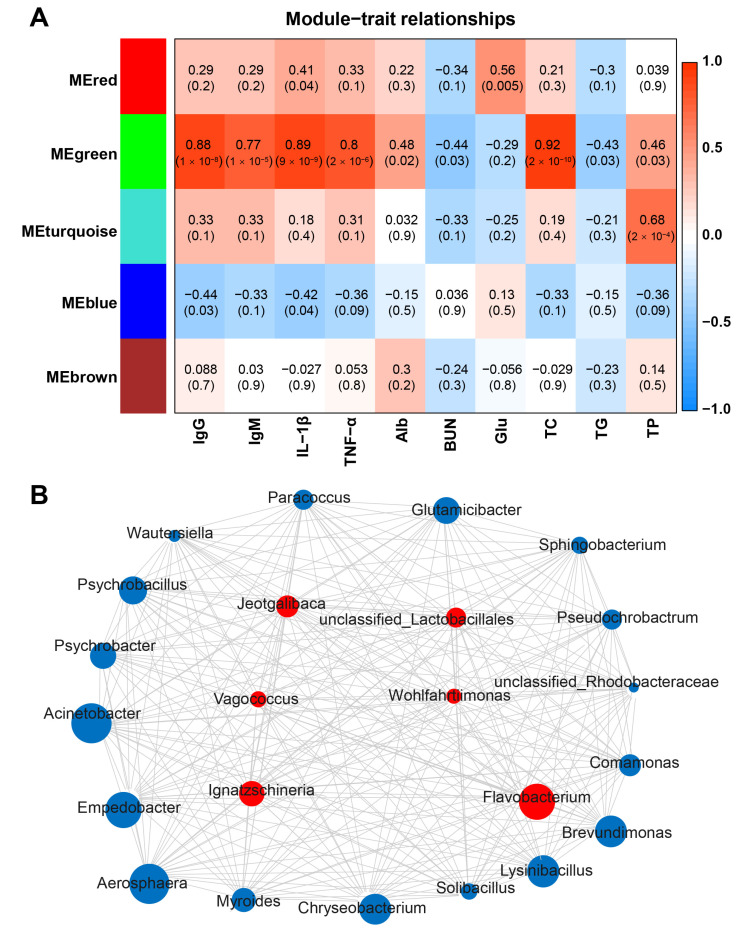
Correlation analysis between the serum immune factors and abundance of the microbiome in feces at the genus level. (**A**) The heatmap of the WGCNA module. (**B**) Immune indexes (IgG, IgM, IL-1β, and TNF-α) of microbial interaction network and hub microbes in the MEgreen module.

At the genus level, the weighted gene co-expression network analysis (WGCNA) was used to explore the association between serum indexes and the microbiota. A total of 5 relevant microbiota modules were identified. The MEgreen module was significantly correlated with immune indexes, including IgG (*p* = 1 × 10^−8^, R = 0.88), IgM (*p* = 1 × 10^−05^, R = 0.77), IL-1β (*p* = 9 × 10^−9^, R = 0.89), and TNF-α (*p* = 2 × 10^−6^, R = 0.80) (Figure 7A). Exporting the network from the MEgreen module showed that *Flavobacterium*, *Ignatzschineria*, unclassified_Lactobacillales, *Jeotgalibaca*, *Vagococcus*, and *Wohlfahrtiimonas* were the hub microbiota in the MEgreen module (Figure 7B). In addition, MEgreen was significantly correlated with TC (*p* = 2 × 10^−10^, R = 0.92). The network derived from the MEgreen module showed that *Acinetobacter*, *Empedobacter*, *Aerosphaera*, *Glutamicibacter*, *Solibacillus*, unclassified_Lactobacillales, *Ignatzschineria*, unclassified_Rhodobacteraceae, and *Wohlfahrtiimonas* were the hub microbiota in the MEgreen module (Supplementary Materials Figure S2).

## 3. Discussion

In this experiment, we analyzed fecal bacterial communities of two small ruminant species to explore the relationship between microbial community structures among different species. A total of 15 bacterial phyla and 866 OTUs were detected. OUTs were more abundant in lambs than in goat kids. In addition, the α-diversity was also significantly different between healthy goat kids and lambs, which was consistent with our results [33]. The results of PCoA analysis showed that different individuals clustered according to species and health status. Previous studies have demonstrated that the microbiota significantly differed between goats and lambs [34] and cows, scalpers, and yaks [35], indicating a species-specific manner in the gut microbiota.

Firmicutes and Bacteroidetes are the two dominant phyla of bacteria in ruminants [36]. Studies in diarrheic calves [37], Yimeng black goats [38], and yaks [39] demonstrated that the gut microbiota was mainly composed of Firmicutes and Bacteroidetes, which was consistent with the results of this study. Our results revealed significant differences in the dominant phyla between HG and HL individuals. However, significant differences were not found in diarrheic animals, indicating that diarrhea impacted gut microbiota composition. In addition, Firmicutes were closely related to fiber degradation in the guts [40]. The abundance of Firmicutes in HL lambs was significantly higher than that in HG goat kids (Figure 1 and Supplementary Materials Figure S1), indicating that the cellulose degradation capacity of lambs was higher than that of goat kids [34].

The gut microbiota is an important marker for evaluating the health status of ruminants and one of the entry points for the treatment of gut-related diseases [41,42]. In calves, the relative abundance of *Negativicutes* in diarrheic feces was higher than in normal feces. At the same time, *Rikenellaceae*, *Tannerellaceae*, *Oscillospiraceae*, and *Anaerovoraceae* were the central gut microbiota of healthy calves [43]. In this study, *Rikenellaceae* and *Oscillospiraceae* were also highly abundant in healthy young small ruminants. In addition, the abundance of Acinetobacter increased in diarrheic yaks [44]. The present study found a similar increasing tendency in diarrheic lambs and goat kids. *Lactobacillus* is considered a probiotic that can improve the gastrointestinal health of pre-weaning calves [45]. In addition, *Lactobacillus* was also used to prevent pathogen colonization in pigs [46]. We also found that *Lactobacillus* was the core bacteria of diarrheic lambs. Furthermore, the proportion of *Lactobacillus* was increased in diarrheic humans, and *Enterobacteria* was reduced after treatment with FMT [47]. This study showed that the proportion of *Lactobacillus* increased and *Escherichia_Shigella* decreased in healthy animals. These findings suggest that microbiota could affect the gut health of animals.

Serum biochemical and immune indexes reflect the physiological health status of animals to a certain extent. Serum immunoglobulin concentration is one of the indexes of the body’s resistance to exogenous pathogenic microbiota [48]. The concentration of IgM and IgG decreased in DL and DG individuals. Meanwhile, we also found a positive correlation between the level of *UCG_005* and IgM. These findings were consistent with a previous study on goats [30]. Most metabolites (Quinethazone, Lithocholic Acid, etc.) were positively correlated with IgG, IgM, IL-1β, and TNF-α. In contrast, Procymidone was negatively correlated with them, which may be related to immune markers and the nature of Procymidone [49]. In addition, diarrhea in mice decreased the IL-1β level in serum [50], and a similar decrease was found in diarrheic lambs and goat kids. We also found that IgG, IgM, IL-1β, and TNF-α were positively correlated with Proteobacteria, *Enterobacteriaceae*, and *Escherichia_Shigella*, which was similar to previous findings [51,52]. IL-1β, TNF-α, and other pro-inflammatory cytokines were correlated with the degree of intestinal inflammation [53]. Moreover, TNF-α, as a pro-cellular inflammatory factor, increased cell permeability, impaired gut barrier function and edema formation, and was associated with diarrhea caused by *E. histolytica* [54,55].

In conclusion, our results characterized the gut microbiota, metabolism, and immune status of lambs and goat kids suffering from diarrhea. Diarrhea led to a decrease in the abundance of Firmicutes in lambs and goat kids. High levels of IgG, IgM, IL-1β, and TNF-α were observed in DL and DG individuals, which were significantly positively correlated with Patescibacteria, *Clostridiaceae*, and unclassified_Muribaculaceae. In addition, the differential metabolites in serum were involved in the bile secretion pathway. Further studies are needed to investigate whether the bacteria or the metabolites identified in this study would improve immunity and prevent diarrhea. Our results provide novel insights into preventing or treating diarrhea in young small ruminants.

## 4. Materials and Methods

### 4.1. Study Location, Samples, and Sampling

A total of 28 lambs, 11 healthy (HG) and 17 diarrheic (DG), and 20 goat kids, 10 healthy (HL) and 10 diarrheic (DL) were used in this study. Goat kids were reared in the National Conservation Farm of Chengdu Brown Goat (Chengdu, Sichuan, China) and the intensive Hu sheep farm (Yulin, Shaanxi, China), respectively. The sampling procedures were approved by the Institutional Animal Care and Use Committee of Sichuan Agricultural University, China (permit no. dky-2020302113). Detailed information about feeding management and sampling was described in the previous studies [12,13]. Briefly, fecal samples were collected from the forty-eight animals using sterile cotton swabs. Simultaneously, two to three milliliters of blood were collected from the jugular on the same day. Blood was clotted at room temperature for 30 min, then centrifuged at 3000 rpm for 15 min at 4 °C to separate the serum. All samples were stored at −80 °C until further analysis.

### 4.2. Gut Microbiome Analysis

To compare the alterations of gut microbiome composition and serum biochemical parameters induced by diarrhea between goat kids and lambs, we reanalyzed the data of 16S rRNA sequencing from our previous published studies [12,13]. In addition, raw data acquisition, quality control, taxonomic information, community composition, the reference Silva database, species classification information corresponding to each feature, and the composition of each sample community were described in the said studies [12,13]. Alpha diversity indices, including ACE, Chao1, Shannon, and Simpson, were estimated using QIIME2-2021.2 [56]. Beta diversity (PCoA) was analyzed for operational taxonomic unit (OTU) clustering based on Bray–Curtis using R software (version 4.2.1). Linear discriminant analysis (LDA) effect sizes were used to detect the dominant bacteria difference between groups. Subsequently, the function of fecal microbiota was predicted using the KEGG database.

### 4.3. Determination of Serum Biochemical Parameters

TP, Alb, Glu, TC, TG, BUN, IgG, and IgM were measured using a Model 7020 automatic biochemical analyzer (Hitachi, Tokyo, Japan). In addition, IL-1βand TNF-α were determined using the ELISA kit (Meimian, China) following the manufacturer’s instructions.

### 4.4. Serum Metabolomics and Data Analysis

The metabolome was determined in the serum samples using the Shimadzu Nexera X2 LC-30AD (Shimadzu, Tokyo, Japan) coupled with Q-Exactive Plus (Thermo Scientific, San Jose, CA, USA). The raw data (Wiff format) were converted to the MzXML format and imported into the XCMS software (version 2.7.2) for peak alignment, retention time correction, and peak area extraction. After the identification of metabolite structures and data pre-processing, the data were qualitatively evaluated. Hotellings T2 and Partial Least Squares Discrimination Analysis (PLS-DA) were used to determine the relationship between metabolite expression levels and sample categories and predict sample categories. Statistically significant metabolites (VIP > 1 and *p* < 0.05) were selected by comparison with the metabolite database. In addition, metabolic pathway enrichment analysis was performed on differential metabolites based on the KEGG database.

### 4.5. Interaction of Gut Microbiome, Host Metabolic and Immune Response

First, the association between microbiota shared with goat kids and lambs at different levels and serum biochemical indexes were analyzed. Then, WGCNA was used to explore the relationship between the microbial composition and serum biochemical indexes, and the network was drawn with Cytoscape (version 3.9.1). In addition, we performed the serum metabolomics of lambs and jointly analyzed them with the metabolomic data of goat kids to assess the interaction of microbiota and metabolites.

### 4.6. Statistical Analysis

The Wilcoxon rank sum test was used to compare the means of groups. Spearman correlation was performed between gut microbiota and serum biochemical indexes. The independent one-way ANOVA test was performed using statistical analysis SPSS version 22.0 software (SPSS Inc., Chicago, IL, USA). LEfSe-calculated scores > 4 were used for LDA to identify bacteria changes in each group. *p* < 0.05 was considered statistically significant. The heatmap was generated using the R program (version 4.2.1) heat map package. Fisher’s exact test was used to identify significantly affected metabolic and signal transduction pathways. The relationship between the immune index and microbiome was investigated using the WGCNA package in R with a soft threshold power of six.

## Figures and Tables

**Table 1:** Lambs and goat kids’ blood biochemical indexes in different health states.

Item	DL	DG	HL	HG	*p* Value	
					DL vs. DG	HL vs. HG
IgM (g/L)	0.087	0.098	0.092	0.103	3.079 × 10^−9^	9.902 × 10^−14^
IgG (g/L)	0.699	0.988	0.763	1.020	4.596 × 10^−20^	7.838 × 10^−21^
IL−1β	65.064	112.521	78.027	161.361	1.038 × 10^−9^	6.836 × 10^−10^
TNF-α	124.148	185.495	146.731	208.137	2.283 × 10^−9^	4.580 × 10^−10^
TP (g/L)	46.609	54.580	44.924	50.180	0.007	0.016
Alb (g/L)	23.573	27.670	21.812	27.170	0.007	3.427 × 10^−6^
Glu (mmol/L)	3.514	3.057	4.001	2.941	0.398	0.014
TC (mmol/L)	1.110	4.286	1.418	5.131	6.624 × 10^−11^	7.876 × 10^−11^
TG (mmol/L)	0.740	0.48	0.340	0.574	0.021	0.004
BUN (mmol/L)	7.454	4.861	5.084	5.958	0.001	0.262

DG: diarrheic goat kids, DL: diarrheic lambs, HG: healthy goat kids, HL: healthy lambs.

## Data Availability

The raw 16S rRNA sequences were deposited in the Sequence Read Archive database under accession numbers PRJNA792435 and PRJNA853133.

## Supplementary Materials

The supporting information can be downloaded at: https://www.mdpi.com/article/10.3390/ijms241411423/s1.
