# Supplementary material for: Combining 16S rRNA Sequencing and Metabolomics Data to Decipher the Interactions between Gut Microbiota, Host Immunity, and Metabolites in Diarrheic Young Small Ruminants
Source: Int J Mol Sci. 2023 Jul 13;24(14):11423. doi: 10.3390/ijms241411423 (PMC10380214; doi:10.3390/ijms241411423)
Supplement: Supplementary file 1 [file ijms-24-11423-s001.zip › Figure S1 and S2.pdf]

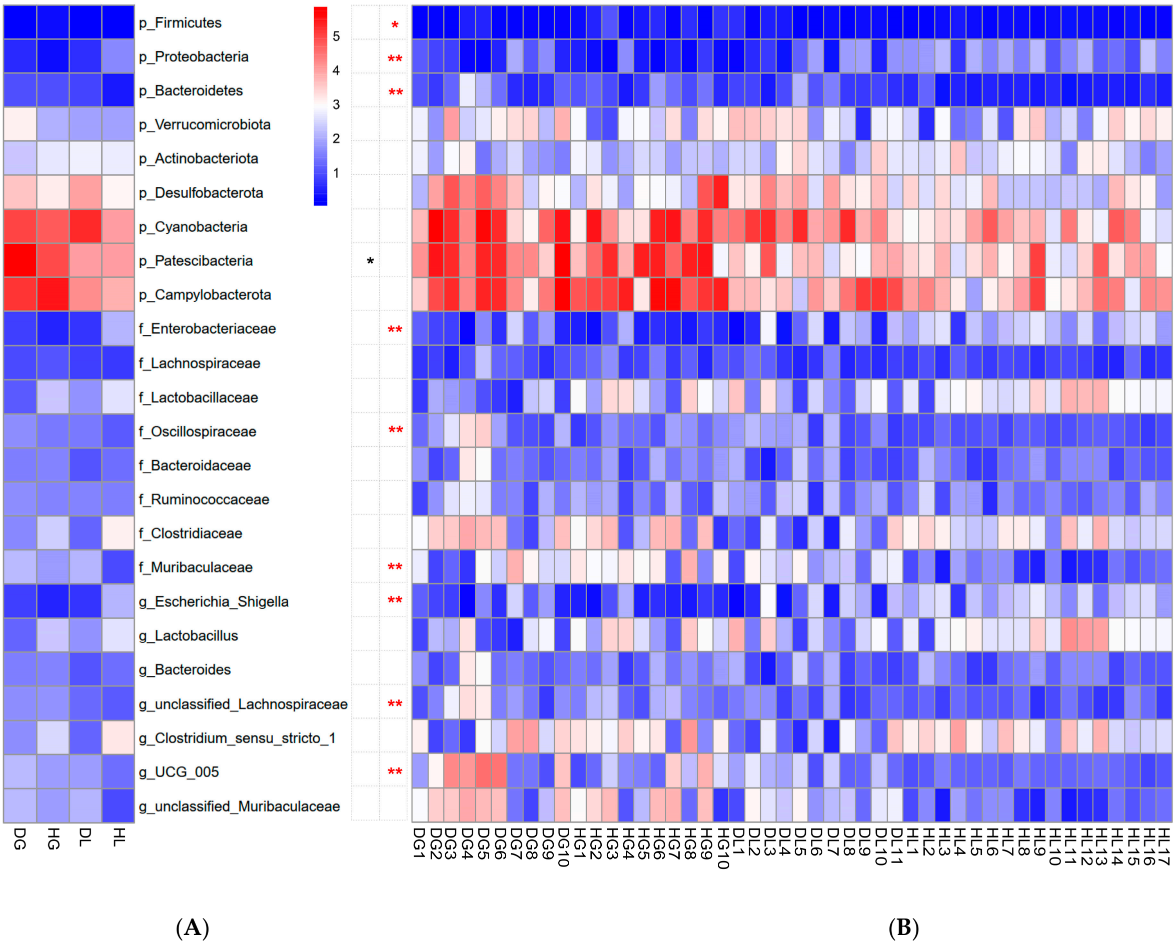

Figure S1. Heat map of the percentage shared microbial community structure in the top 10 at the phylum, family, and genus level on goat kids and lambs, respectively. (A) Four groups. (B) Each sample of four groups. All data are converted by -log10. The left side represents DL versus DG, the right represents HL versus HG. * $p < 0.05$; ** $p < 0.01$.

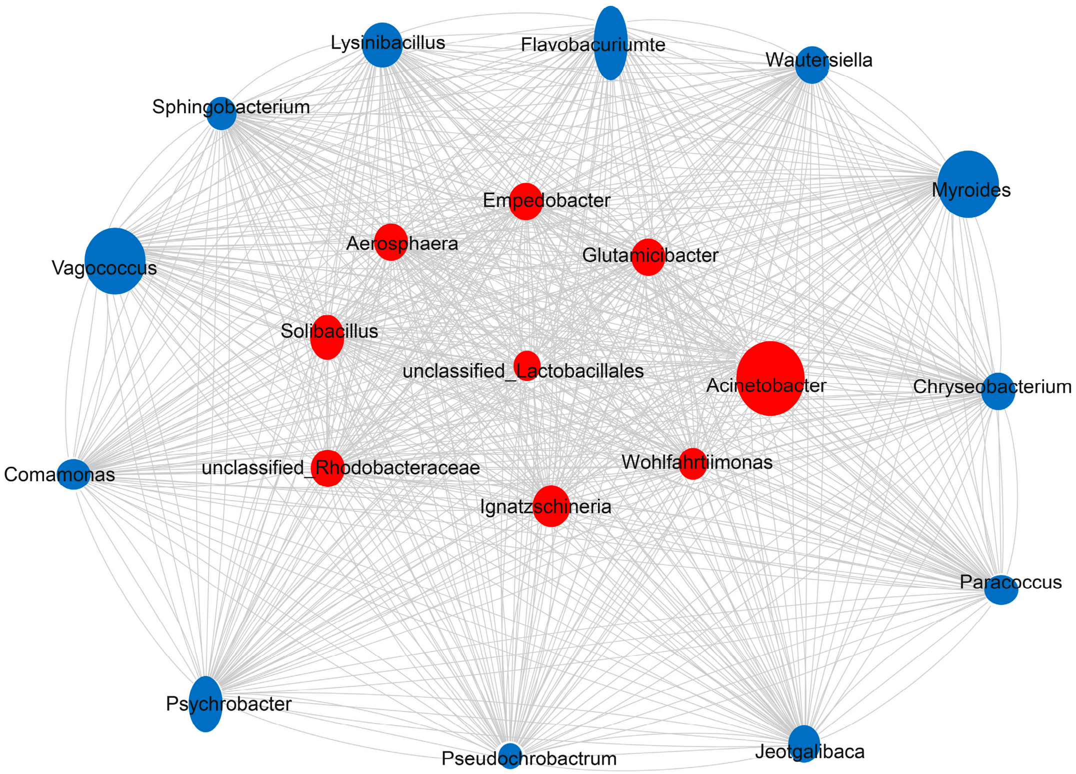

Figure S2. TC–microbial interaction network and hub microbiota in the MEgreen module.
